# Traumatic obturator dislocation of the hip joint: a case report and review of the literature

**DOI:** 10.11604/pamj.2015.21.55.6392

**Published:** 2015-05-25

**Authors:** Redouane Hani, Mohamed Kharmaz, Mohamed Saleh Berrada

**Affiliations:** 1 Departement of Orthopedic Surgery, Ibn Sina Hospital, Rabat, Morocco

**Keywords:** Hip dislocation, obturator, closed reduction, osteonecrosis

## Abstract

We describe a case of traumatic obturator hip dislocation in an adult. Closed reduction was done under general anesthesia. Post-reduction radiographs showed concentric and congruent reduction of the right hip. Traction was applied for three weeks followed by progressive mobilization and loading. Follow up for two years after the injury showed that the patient achieved a full recovery without any evidence of hip pain or a decreased range of motion. There were no signs of osteonecrosis of the femoral head.

## Introduction

The rise of road traffic accidents involving high-energy trauma has increased the incidence of traumatic hip dislocation. Obturator hip dislocations in adults are rare, and only a few cases have been reported in the literature. We describe an adult case of traumatic true obturator hip dislocation.

## Patient and observation

**Figure 1:**
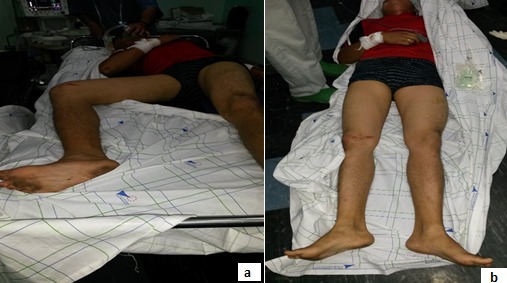
Closed reduction of the right hip joint under general anesthesia: (a) before reduction; (b) after reduction

Male patient, 35-years-old, victim of an automobile accident was admitted in our emergency department two hours after. He complained about severe pain in his hip and inability to move the right lower limb. On physical examination he was conscious and hemodynamically stable, the lower limb was found in extension; abduction and external rotation. There were no neurovascular deficits without associated injuries Figure 1.

**Figure 2:**
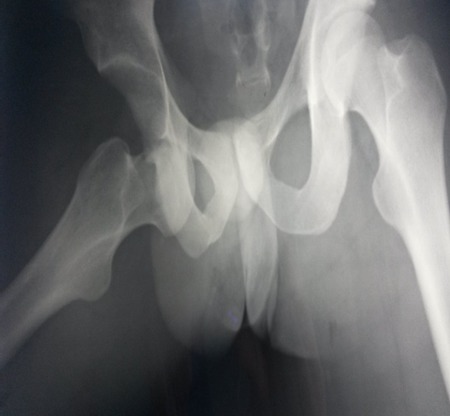
Anteroposterior radiograph of the pelvis showing an obturator dislocation of the right hip

**Figure 3:**
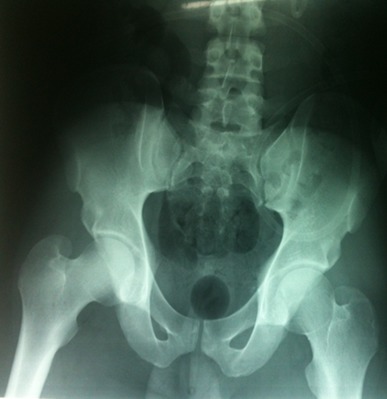
Post reduction radiograph showing the right hip congruency

**Figure 4:**
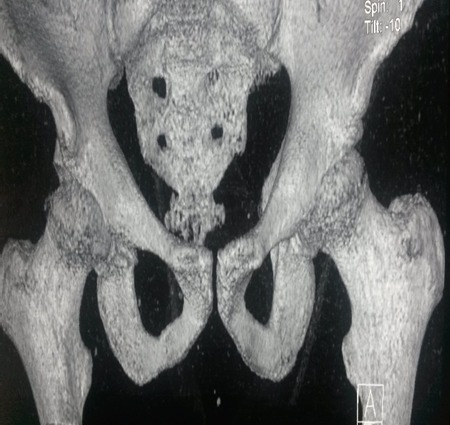
Post reduction CT showing no fracture of the femoral head

Radiographic examination of the pelvis revealed an obturator dislocation of the right hip. No associated fracture was seen Figure 2. The dislocation was immediately reduced under general anesthesia by traction in the line of the deformity followed by a gentle adduction and internal rotation, the pelvis was stabilized by an assistant. Clinical and radiographic evaluation showed a stable reduction Figure 3. Computed tomography showed no fracture of the femoral head. The patient was kept on bed rest for 3 weeks with continuous traction and he was permitted full weight bearing 6 weeks after the injury Figure 4.

Follow up for two years after the accident showed that the patient was pain free with full range of motion. There were no changes suggestive of avascular necrosis of the femoral head.

## Discussion

Anterior dislocations of the hip are divided into two types according to the position of the femoral head, pubic or superior (type 1) and obturator or inferior (type 2) [1, 2].

Obturator dislocations of the hip are uncommon injury, occurring in less than 5% of all traumatic hip dislocations [2, 3]. The lower incidence of anterior dislocation may be due to in part to the strong anterior capsule and the Y-shaped ligament of Bigelow [1]. They occur as a result of the forced abduction, external rotation and flexion of the hip joint [4]. Road traffic accidents were responsible for the majority of anterior obturator dislocations of the hip with dashboard impact, where sudden deceleration created the dislocating force [5].

Dislocation of the hip is an orthopedic emergency. Closed reduction under general anesthesia is considered as the treatment of choice in traumatic obturator hip dislocations [6, 7]. Reduction must be performed within 6 hours after trauma to reduce the risk of avascular necrosis witch is seen in 50% of cases if the hip is reduced more than six hours after the injury [1, 2]. In our case, the reduction was done within four hours after the accident. Obturator hip dislocation witch is irreducible by closed reduction requires open reduction through an ileo-inguinal approach. Toms et al reported a case of open reduction with release of the rectus femoral muscle [8]. Traction has been recommended for three to six weeks after the hip dislocation to allow capsular healing, followed by progressive mobilization and loading [9, 10]. Suitable imaging is needed to exclude indentation fractures and to monitor the vascularity of the femoral head.

## Conclusion

Obturator dislocation of the hip in adults is rare. Its rarity is due to the inherent stability of the joint, its deep position in the pelvis with strong ligaments and bulky muscles around the articulation. Prompt diagnosis and treatment are crucial in the management of these injuries.
